# Multi-Layer Technique (MLT) with Porcine Collagenated Cortical Bone Lamina for Bone Regeneration Procedures and Immediate Post-Extraction Implantation in the Esthetic Area: A Retrospective Case Series with a Mean Follow-Up of 5 Years

**DOI:** 10.3390/ma14185180

**Published:** 2021-09-09

**Authors:** Paul Leonhard Schuh, Hannes Wachtel, Florian Beuer, Funda Goker, Massimo Del Fabbro, Luca Francetti, Tiziano Testori

**Affiliations:** 1 Private Practice, 80803 Munich, Germany and Charité Berlin, Department of Prosthodontics, Geriatric Dentistry and Craniomandibular Disorders, 14197 Berlin, Germany; mail@paulschuh.com; 2 Implaneo GmbH MVZ Dental Clinic, 81679 Munich, Germany and Charité Berlin, Department of Prosthodontics, Geriatric Dentistry and Craniomandibular Disorders, 14197 Berlin, Germany; hannes@wachtel.biz; 3 Charité Berlin, Department of Prosthodontics, Geriatric Dentistry and Craniomandibular Disorders, 14197 Berlin, Germany; florian.beuer@charite.de; 4 Department of Biomedical, Surgical and Dental Sciences, Università degli Studi di Milano, 20122 Milan, Italy; funda.goker@unimi.it (F.G.); luca.francetti@unimi.it (L.F.); info@tiziano-testori.it (T.T.); 5 IRCCS Orthopedic Institute Galeazzi, Dental Clinic, 20161 Milan, Italy; 6 Department of Periodontics and Oral Medicine, School of Dentistry, University of Michigan, Ann Arbor, MI 48109, USA

**Keywords:** alveolar reconstruction, collagenated cortical bone, dental implants, edentulous patient, anterior maxilla, anterior mandible, porcine bone, retrospective study, multi-layer technique

## Abstract

Background: Augmentation of the edentulous atrophic anterior region is a challenging situation. The purpose of this article was to evaluate the effectiveness of a collagenated cortical bone lamina of porcine origin for horizontal ridge augmentation in patients with inadequate alveolar ridge width undergoing immediate post-extraction implantation in the anterior sites, and to report on implant survival rates/complications. Materials and methods: The cases were extracted electronically from a large database according to these specific inclusion criteria: patients with inadequate alveolar ridge width in the anterior maxilla or mandible, who underwent immediate post-extraction implant placement and simultaneous alveolar bone reconstruction using xenogeneic cortical bone lamina. An additional layer of palatal connective tissue graft was inserted between lamina and the vestibular mucosa, for improving soft tissue healing. A collagenated bone substitute was additionally placed in the gap between the lamina and implant surface in all patients. The main outcomes were implant survival and complications. Results: Forty-nine patients with 65 implants were included. Patients’ mean age at the time of implant surgery was 60.0 ± 13.6 years. The mean follow-up was 60.5 ± 26.6 months after implant placement. The implant survival was 100%. Four postoperative complications occurred in four patients. No specific factor was found to be associated with complication occurrence. Conclusion: The use of collagenated cortical bone lamina can be considered as a successful option for alveolar reconstruction in immediate post-extraction implant insertion procedures in anterior regions with inadequate alveolar ridge width.

## 1. Introduction

Reconstruction of atrophic jaws is still a challenging situation for surgeons [1,2], especially in the esthetic area where the treatment of anterior defects is essential to restore function and esthetics of the whole face [3,4]. There are various options described in literature for placing implants immediately after tooth extraction with successful results [2,3,4]. However, there is currently no established gold standard, nor a universal consensus, on which option is best. Disagreement still exists in the methods suitable to address all the problems associated with such clinical situations [1,2]. Decisions should always be related to several factors depending upon the hard and soft tissue conditions after tooth extraction which might affect the complexity of the procedure, and successful outcomes.

Adequate integrity and volume of the alveolar bone are fundamental to successful implant-supported rehabilitation in any clinical situation, and a diversity of grafting materials were proposed in literature to re-establish the lost functions and esthetics [5,6,7,8,9,10,11]. Among these options, xenogeneic bone substitutes are among the most popular methods due to many advantages such as osteoconductivity and avoidance of donor site morbidity [9,10,11]. Xenografts that could be either collagenated (fully resorbable) and non-collagenated (more similar to ceramic biomaterials with a very slow resorption rate) are valuable as bone substitutes because they show similar bone morphology with human bone, and can be used in conjunction with collagenated materials [12,13,14].

Collagenated cortical bone lamina of porcine origin is a recently developed material with biological and mechanical properties favorable to successfully achieving hard tissue augmentation [15]. The main purpose of this clinical case series report was to evaluate the prognosis of a novel clinical approach called the multi-layer technique (MLT), consisting of soft tissue graft, collagenated porcine cortical lamina, and a porcine particulated graft material in patients undergoing immediate implantation in the anterior post-extraction sites with missing socket buccal bone, and to report survival rates and complications, over a five-year follow-up period.

## 2. Materials and Methods

This multicentric retrospective case series study was carried out in private clinics and consisted of patients that had ridge augmentations with cortical bone lamina, simultaneous to immediate implant placement in postextraction sockets. All the patients were treated and followed between February 2012 and December 2020. The study was in compliance with the principles laid down in the Declaration of Helsinki on medical protocol and ethics. A signed informed consent form was obtained from all subjects for the medical and surgical procedure and for the use of data in the research. Institutional Review Board approval of the IRCCS Orthopedic Institute Galeazzi was obtained for retrospective studies on implant therapy, with number 2552377-L2058/RC 2019.

### 2.1. Patient Selection

Medical records were collected retrospectively from the clinics’ databases of patients who had bone augmentations with a multi-layer technique involving the use of cortical bone matrix (OsteoBiol^®^ Lamina Soft, Tecnoss^®^, Giaveno, Italy), and received one or more dental implants for oral rehabilitation in the anterior maxilla.

The inclusion criteria for treatment were as follows:

- Patients older than 18 years of age;
- Patients without any general medical contraindications for grafting procedures;
- Patients physically able to undergo surgical interventions: American Society of Anaesthesiologists ASA-1 or ASA-2; and
- Patients with a missing buccal plate, in need of tooth extraction, in which the alveolar ridge was deficient in the buccolingual dimension (<5 mm), who required a lateral ridge augmentation for implant placement.

Smoking habits, controlled diabetes, osteoporosis, bruxism and minor systemic conditions were not considered as exclusion criteria.

The implants utilized in this study consisted of Nobel Active, Nobel Replace CC, Nobel Speedy Groovy, Nobel Parallel CC (Nobel Biocare, Kloten, Switzerland), Straumann BLX (Straumann, Andover, MA, USA), GM Helix (Straumann, Andover, MA, USA), 3i OsseoTap Certatin (Zimmer Biomet, Palm Beach Gardens, FL, USA). The implants were inserted immediately after extractions of teeth in the same surgical session with bone augmentation, with OsteoBiol^©^ Lamina Soft (Tecnoss^®^, Giaveno, Italy) and bone substitute (OsteoBiol^®^ mp3^®^, Tecnoss^®^, Giaveno, Italy). All the implants were placed according to the manufacturers’ surgical recommendations.

Prior to therapy, all patients were radiologically evaluated either with panoramic radiograph, periapical X-rays or CBCT. A detailed anamnesis of general health status was also performed by clinical intra/extra-oral examination. Patient preparation consisted of oral hygiene instruction several weeks before the surgical procedure, and in cases of poor oral health, a professional oral hygiene session was scheduled.

### 2.2. Surgical Technique

Starting from a day before surgery, prophylactic antibiotic was prescribed to each patient, with Augmentin (amoxicillin and clavulanate potassium) at a dosage of 1 g tablet every 12 h or Azithromycin 500 mg every 12 h in cases of allergy to penicillin. On the day of surgery, after rinsing with a 0.2% chlorhexidine-digluconate solution and application of local anesthesia (4% articaine with 1:100,000 adrenalin), an intrasulcular incision was made around the tooth with a #15c surgical scalpel. The site was additionally prepared with periotomes and microlevers for a minimally invasive dental extraction procedure, and the tooth was extracted with maximum attention. Following the extraction, for removal of the granulation tissue and the residual of the periodontal connective tissue, a careful curettage of the socket was performed.

At this point, if immediate implantation was planned, the implant site was prepared using drills and burs according to the instructions from the manufacturer. The implant was inserted with final insertion torque of 50 Ncm, with palatal anchorage. The next step was the augmentation of the buccal aspect of the tooth socket by a multi-layer technique. This technique consisted of the apposition of three distinct materials: a free soft tissue graft from the same patient, collagenated cortical bone lamina matrix (OsteoBiol^®^ Lamina Soft, Tecnoss^®^, Giaveno, Italy), and collagenated porcine bone substitute (OsteoBiol^®^ mp3^©^, Tecnoss^®^, Giaveno, Italy).

In brief, the surgery continued with soft tissue graft harvesting from the palatal site of the patient. The graft was de-epitalized, placed inside the extraction socket and fixed to the buccal mucosa by means of two horizontal mattress sutures. Next, the cortical lamina bone graft was inserted between the soft tissue graft and the implant. In this study, two models of soft lamina were used: 1/Code LS25FS OsteoBiol^®^ Lamina Soft, Fine, Porcine, 25 × 25 × 0.5 mm (cortical flexible lamina); 2/Code LS10HS OsteoBiol^®^ Curved soft Lamina, Porcine, 35 × 35 × 0.9 mm (curved cortical flexible lamina). The choice among them depended on the preferences of the surgeon in terms of handling and thickness, and according to the defect characteristics. Prior to use, the cortical bone matrix (OsteoBiol^®^ Lamina Soft, Tecnoss^®^, Giaveno, Italy) was hydrated in sterile saline for 5/10 min. Once the Lamina Soft had reached the desired plasticity, it was cut, adapted and stabilized as a second layer over the soft tissue graft. The stabilization of the cortical lamina bone graft was achieved at the time of insertion, since the graft was modeled to adequately fit the defect dimensions.

Subsequently, a collagenated porcine bone substitute (OsteoBiol^®^, mp3^©^, Tecnoss^®^, Giaveno, Italy) was inserted and compacted between the lamina and the surface of the implant. Condensation of the particulated bone substitute was made in small increments, from an apical to a cervical direction, with caution to avoid graft dislocation. For this purpose, a small bone compactor (Schwert, Seitingen-Oberflacht, Germany) was used in the apical region and a larger diameter bone compactor was used in the more coronal region. Lastly, the provisional crown was installed over the implant with attachment screw (with 20 Ncm torque), allowing sealing of the gingival margin for protection of the multi-layered graft. In case of delayed loading, the provisional crown was attached to the adjacent teeth by using light-cured composite fillers.

### 2.3. Post-Operative Protocol

After surgery, patients were instructed to avoid any load on the surgical site and to perform topical application of 0.12% chlorhexidine gluconate (PerioGard^®^, Colgate-Palmolive, Hamburg, Germany) for seven days, twice a day, followed with 0.2% chlorhexidine di-gluconate mouth rinses three times daily for at least two weeks. In order to reduce swelling, ibuprofen 600 mg every 12 h for five days was prescribed. Oral antibiotic therapy was prescribed to all patients, consisting of amoxicillin and clavulanate potassium 1 g every 12 h for five days, or azithromycin 500 mg every 12 h for five days as an alternative in case of allergy to penicillin.

### 2.4. Follow-Up

The sutures were removed 8–10 days later and standard follow-up visits, including clinical examinations were scheduled on regular basis at 1 month, 3 months, 6 months, 12 months; and then, every 6 months for the following years. All the patients received their final/temporary prosthesis as planned, using the manufacturer’s components or cemented on customized abutments.

### 2.5. Outcomes

Implant survival was considered as the primary outcome of the study. The intra-surgical and post-surgical complications were assessed as outcomes. The clinical follow-up examinations were done by the same surgeon who had performed implant surgery. Implants were considered to be successful according to the following criteria:

- implant that is still functional supporting a prosthetic restoration;
- healthy peri-implant tissues;
- absence of clinical mobility;
- no radiographic evidence of peri-implant radiolucency; and
- absence of any post-operative complications.

For each patient, the parameters listed in Table 1, Table 2, Table 3 and Table 4 were collected.

### 2.6. Definition of Peri-Implant Mucositis and Peri-Implantitis

Peri-implant mucositis: presence of bleeding on probing (BoP+) and/or inflammation.

Peri-implantitis: BoP+ and/or suppuration, increasing bone loss compared to the baseline (crown delivery).

Peri-implant tissues were considered as healthy when there was neither mucositis nor perimplantitis.

### 2.7. Statistical Analysis

Descriptive statistics of the data was done using mean values and standard deviation (SD) for quantitative variables normally distributed; in addition, 95% confidence intervals were estimated. Normality of distributions was evaluated through the D’Agostino and Pearson omnibus test. The effect of variables on complications was evaluated by using the Fisher’s exact test. The unit of analysis was the implant. *p* = 0.05 was considered as the significance threshold. Statistical analysis was performed using GraphPad Prism version 5.03 (GraphPad Software, Inc., La Jolla, CA, USA).

## 3. Results

A large personal database of patients was evaluated, and patients were extracted electronically according to the inclusion criteria strategy described in materials and methods.

The initial research in patient population data in the office management software since 2010 resulted in 28,507 patients. Soft lamina applications (757 patients) were extracted from these patients. There were 459 patients with “6311040 Lamina Oval” applications, plus 322 patients with “6311020 Lamina Dried” (Group A), and 1078 patients with implants and CTG (Group B).

The overlap of Group A and B resulted in 255 patients.

After applying exclusion criteria (1. All-on-implant cases; 2. All cases where the implants were in the posterior region; 3. Cases where the augmentation with the bone lamina or the soft tissue and the implantation were in different areas), 177 patients were excluded. Finally, only patients undergoing immediate implantation in postextraction sites were selected.

As a final result, a total of 49 patients and 65 implants with a mean follow-up of 60.5 ± 26.6 months after implant placement (mean 53.5 months after loading) were included in this study. The mean age of the patients at the time of implant surgery was 60.0 years (SD 13.6, range 31–93 years). Thirteen patients (18 implants) were smokers. Periapical infection was present in 53 teeth. Thirty-six teeth had previously undergone root canal treatment. Fifty-seven implants were placed in the anterior maxilla, and eight in the anterior mandible. In 34 patients (49 implants), all surgical procedures were done in the same surgical session, and no further surgery was required, while 15 patients (16 implants) underwent two surgical sessions. In eight patients (nine implants), the second session was necessary to perform an additional connective tissue graft, in order to optimize the vestibular contour. Immediate loading was performed in 40 cases, while in 25 implants the functional loading was applied after a mean period of 8.8 ± 4.5 months. Forty-one patients (57 implants) had screwed prosthesis, while eight (eight implants) had cemented prosthesis. Table 1 shows general characteristics of the patient population including age and smoking habits. In Table 2, characteristics of extracted teeth, implant insertion and surgical procedure are described. No implant was lost throughout the observation period, leading to an implant survival of 100%. Four post-operative complications occurred in four patients (listed on Table 3), while 45 patients had no complications. All complications were promptly addressed and had no consequences on the treatment effectiveness. Table 4 reports the synthetic results of the analysis of factors potentially affecting the incidence of complications. No factor among those examined was found to be related to post-surgical complications.

**Figure 1:**
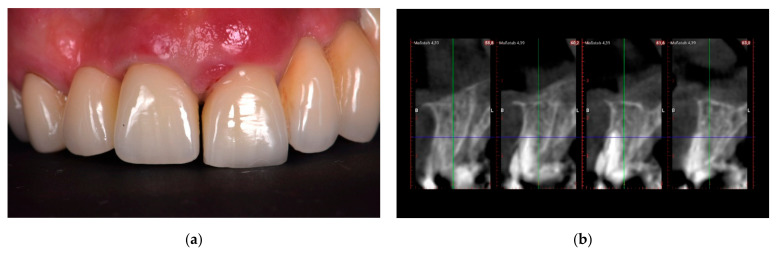
First clinical case. Pre-operative intra-oral view of a patient: (**a**) frontal view of the compromised tooth #21; (**b**) pre-operative CBCT radiographs.

**Figure 2:**
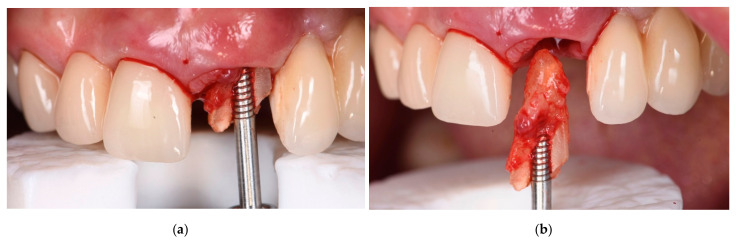
(**a**,**b**). Intra-operative view of the atraumatic extraction procedure of tooth #21.

**Figure 3:**
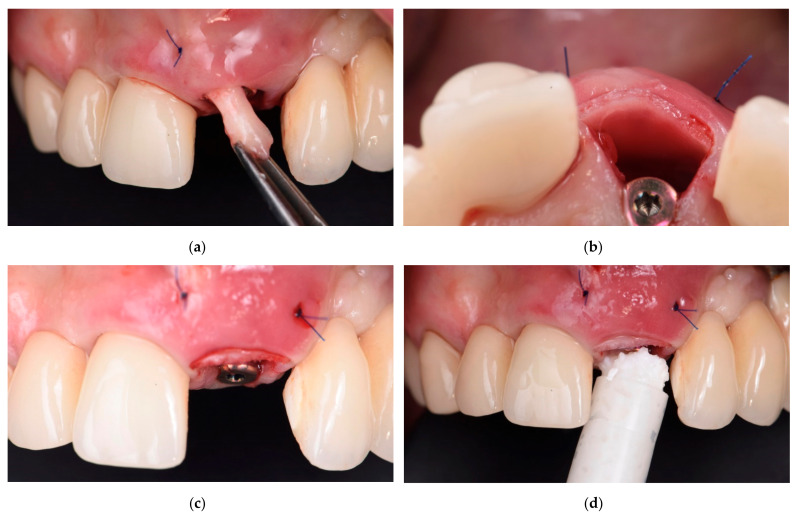
Intra-oral view of the patient showing the steps of the three-layer protocol after implant placement: (**a**) placement of the harvested free soft tissue graft; (**b**) Soft tissue graft was stabilized with two horizontal mattress sutures, and cortical lamina graft was inserted and put in contact with soft tissue graft. Note the gap between the cortical lamina graft and implant; (**c**) frontal view showing implant, soft tissue and lamina graft in place; (**d**) placement of the particulated bone graft (OsteoBiol^®^ mp3^®^) into the gap between implant and lamina graft.

**Figure 4:**
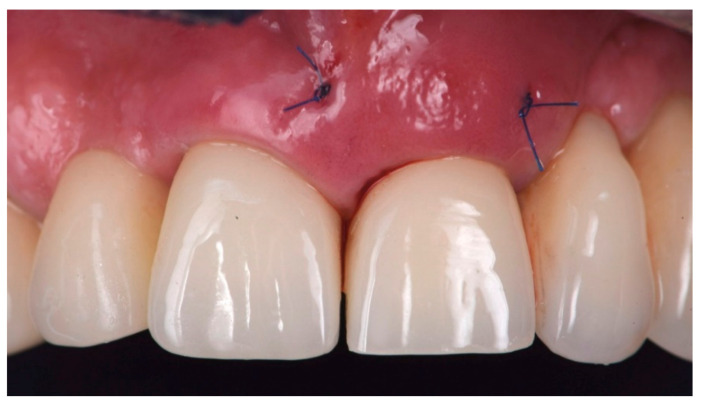
Intra-oral view of the patient, showing the provisional crown installed over the implant with attachment screw, allowing sealing of the gingival margin for protection of the multi-layered graft.

**Figure 5:**
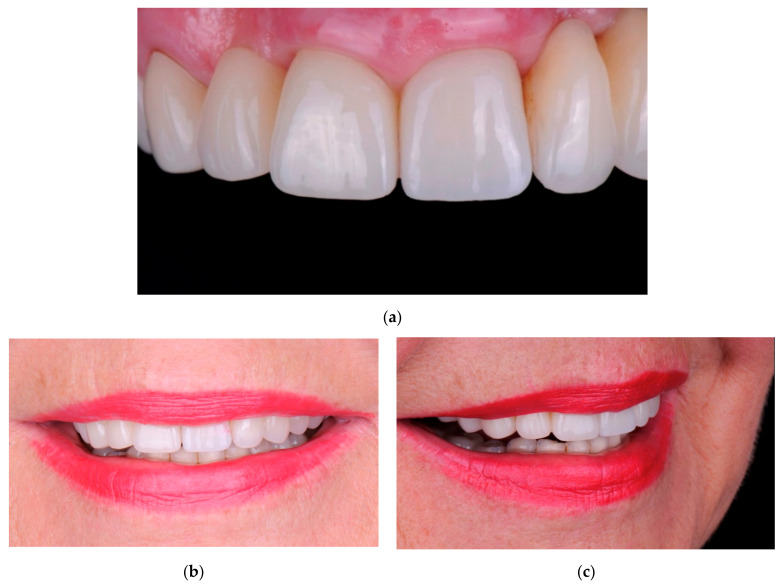
Images of the final case after 12 months follow-up. (**a**) Intra-oral view of the patient showing the final crown; (**b**,**c**) Extra-oral photographs of the patient.

One complete clinical case, illustrating the steps of the multi-layer procedure for post-extraction site reconstruction, is documented in Figure 1, Figure 2, Figure 3, Figure 4 and Figure 5.

## 4. Discussion

Healing of bone defects, following the use of bone grafting materials, depends greatly on the interaction between the bone graft and the bone cells of the host [16,17]. The factors that are influencing the bone regeneration are the defect morphology, physico-chemical properties of the biomaterials and bone regenerative potential of the patients [16,17].

In a systematic review, Esposito et al. evaluated the horizontal and vertical bone augmentation techniques; as a conclusion, no protocol was found more efficient among the options [18]. Among the alternatives, combination of xenografts with autogenous bone and the use of titanium reinforced polytetrafluoroethylene (PTFE) membranes is one of the most applied, with successful results but also a high incidence of complications [19,20].

The bone lamina technique represents a peculiar solution for augmentation of edentulous alveolar ridges, with mechanical properties analogue to titanium membranes, but also with specific features, including full biocompatibility. The cortical lamina is a collagenated porcine bone graft barrier made of cortical bone of heterologous origin, which is produced with an exclusive process, and consists of three distinct products: Lamina Soft, Curved Soft Lamina, and Rigid Lamina. The exclusive process by the manufacturer avoids the ceramization of hydroxyapatite crystals, so that physiological resorption of the lamina barrier is accelerated. Following a process of superficial decalcification, the Lamina Soft achieves an elastic consistency, yet preserving the typical compactness of the bone tissue from which it originates. The borders of the product are soft, so as to avoid micro traumas to the neighboring tissues. It has properties such as gradual resorption, a rigid, moldable, and adjustable membrane that is actually made of bone, and it adapts well to local anatomy in edentulous mandible or maxilla. In fact, it can be shaped using sterile scissors, until the necessary size is reached, then it has to be hydrated for 5–10 min in sterile saline (0.9% NaCl) solution. Once it achieves the desired pliability, it can be shaped and applied to the defect site. It can be fixated with pins or osteosynthesis screws [13,15,21], or directly sutured (the Fine model) to the surrounding tissues, with a triangular section non-traumatic needle [22]. These features are particularly helpful when it is necessary to maintain the graft volume in aesthetic areas, as well as in horizontal augmentation [15,23,24] of two wall defects, and for covering antrostomy in lateral access sinus augmentation procedures [21,25,26]. Lamina can also be used in regeneration procedures with the risk of exposure. Curved Soft Lamina has a semi-rigid consistency, has a thickness of about 1.0 mm, and must be directly grafted without hydration, after being shaped to fit the defect morphology [27]; it can be particularly effective in association with bone substitutes for regeneration of ridges in which the buccal plate is compromised. Rigid Lamina has been documented for orbital floor and wall reconstruction [28]. The recently developed 0.7 mm thickness Rigid Lamina undergoes a process of superficial semi-decalcification (about 50% as compared to Lamina soft), therefore increasing its consistency, typical of the cortical bone tissue [29,30]. It may represent a viable alternative to autogenous cortical bone plates in the reconstruction of three-dimensional crestal defects with the shell technique [31].

Currently, publications on lamina are limited in number, however they show quite promising outcomes [13,27,32,33]. A multi-center study evaluated the efficacy of the lamina in protecting grafted defects over implants [13], while others tested the cortical lamina functioning as a membrane when treating horizontal defects, and both reported excellent clinical, biological and esthetic outcomes [32,33]. More recently, lamina was tested in the posterior atrophic mandibular bone in a clinical and histological study [27]. As a result, all of the implants were osteo-integrated, and after one year of loading there was no bone loss at the crestal level around the implants. Biopsies showed new formation of bone in the areas that were augmented with lamina.

The technique described in this study is a technical modification of a protocol that was originally described by da Rosa et al. in 2013 [34] for immediate dentoalveolar restoration of compromised sockets in a case report. The proposed treatment by da Rosa et al. [34,35] followed a protocol of immediate implantation, with a flapless surgery, and using corticocancellous bone graft harvested from the maxillary tuberosity to restore the bone defect [34,35]. The same team of authors additionally evaluated the esthetic outcomes and tissue stability of this protocol in a prospective study which included 18 patients with a mean follow up of 58 months, and reported promising outcomes [36]. In that prospective study, the patients had their teeth scheduled for extraction and immediate implantation. In brief, the technique by da Rosa et al. included the following steps: the tooth was extracted as atraumatically as possible, the socket was carefully curetted to remove any granulation tissue, dental implants were inserted in the cingulum axis of the extracted tooth, autogenous corticocancellous bone graft was harvested from maxillary tuberosity, the graft was shaped for adaptation to the defect site, the graft was carefully inserted into the defect between the implant and the mucosal tissue, particulate cancellous bone was inserted and packed to fill the spaces between the corticocancellous bone graft and the implant surface, and subsequently, a provisional crown restoration was placed onto the implant [34,35,36].

A similar protocol was also assessed by various authors with favorable results [37,38,39]. The chamber concept explained by Degidi et al. included immediate provisionalization of implants placed in fresh extraction sockets using a definitive abutment [37]. In that paper, ten patients were treated with immediate flapless extraction, implant placement, grafting with bone substitutes into the gap between the inner surface of the buccal wall and the implant surface, and the provisional crown was placed on the standard abutment [37]. De Molon et al., in 2015 [38], evaluated results of reconstruction of the alveolar buccal bone plate in compromised fresh socket after immediate implant placement followed by immediate provisionalization [38]. De Molon et al., in that case report in 2013, reported a hopeless maxillary left central incisor with loss of the buccal bone wall, which was treated with flapless extraction, immediate implant placement, and immediate reconstruction of the buccal bone plate using the tuberosity as the donor site (to obtain block bone and connective tissue grafts, as well as particulate bone). For this purpose, an autogenous block bone graft and connective tissue graft were harvested from the maxillary tuberosity. After reshaping to match the bone defect, the graft was placed in position with the connective portion facing the gingival mucosa, and the bone portion facing the implant. Subsequently, the particulate autogenous bone graft was placed to completely fill the remaining gap between the implant and the bone graft, followed by the placement of an immediate provisional restoration [38].

In a more recent report by Sheejith et al. in 2020, the “ice cream cone technique” was represented as a flapless bone regeneration technique [39]. In fact, that technique (“ice cream cone”) was originally invented by Tarnow to augment the socket with buccal dehiscence, but his technique was only recommended for simple dehiscence and not a wall defect [40]. In that technique, a collagen membrane in the form of an ice cream cone was used and bone filler material placed into it, to regenerate the buccal plate of a fresh socket without elevating a flap [39,40]. In brief, the tooth was extracted, collagen membrane was contoured into a modified V shape/ice cream cone shape and was placed into the socket, wide enough to extend laterally. The socket was filled with a bone graft with the top part of the membrane extended over the opening of the socket, and the membrane was then sutured to the palatal tissue. As a result, they reported no change in the mucogingival junction position, and prevention of invasion of soft tissue into the socket. However, as a disadvantage, there was a decrease in the depth of the buccal vestibule at the end of healing period, and difficulty in placing the membrane as it became softened when exposed to fluid [39].

The novelty of the multi-layer protocol presented in this paper is the three-layer method used for grafting: a free soft tissue flap that was obtained from the palatal site of the same patient, cortical bone lamina matrix (OsteoBiol^®^ Soft Cortical Lamina, Tecnoss^®^, Giaveno, Italy), and the collagenated porcine bone substitute (OsteoBiol^®^ mp3^®^, Tecnoss^®^, Giaveno, Italy). The cortical lamina used was easy to handle and shape for a perfect adaptation for the full coverage of the extraction defect, and volume maintenance. Utilization of soft tissue graft, together with cortical lamina and particulate bone substitute ensured the good esthetic results. Furthermore, the multi-layer technique has several advantages:

- There is no donor site, and this reduces the invasiveness of the procedure;
- it is more versatile, since it can be performed even in patients who still have upper wisdom teeth, compared to the original technique, which can be done only if the wisdom teeth have been extracted; and
- the connective tissue graft could potentially make the soft tissue more stable in the long term.

The absence of radiological evaluation of marginal bone level change could be considered a limitation of this study. However, as the reconstruction procedure herein presented was associated with immediate implantation and mostly immediate loading, it is very challenging to identify a baseline peri-implant bone level for which to refer in subsequent radiographic assessments.

According to the results of the present study, lamina technique is a successful option to treat anterior defects, which represent a unique and challenging situation in the dental practice, especially in the maxilla. Bone lamina always integrated in a biologically correct way, serving as a semi-rigid barrier for the reconstruction of the resorbed alveolar ridge. Bone lamina contributed to the correct regeneration of the missing bone volumes, and as a result, patient esthetics, function and satisfaction was high in the study population. The post-operative complications were minor and few in number.

## 5. Conclusions

Based on the results of this study, the following conclusions can be drawn:

1. The present multi-layer technique using bone lamina can be considered as a successful and valuable option with mid-term follow-up up to five years for immediate postextraction implants in sockets with a missing buccal wall.
2. There are some limitations due to the retrospective nature of the study.

Future prospective studies, aiming at quantifying bone regeneration, bone level changes, and patient-related outcome measures, are needed to confirm the excellent clinical results herein observed.

## Figures and Tables

**Table 1:** General anamnesis results of patient population including age and smoking habits.

Pat. No.	Age at Surgery, Years	Smoker–n. Cigarettes/day	General Anamnesis, Drugs Taken
1	51	yes-10/d	Non-contributory
2	78	no	Bypass—Ramipril, Clopidogrel
3	72	no	Breast cancer, 2010—Votum, Toroxin 100
4	49	yes-20/d	Non-contributory
5	63	yes-20/d	Fosamax (single shot)
6	57	yes-10/d	Non-contributory
7	46	yes	Non-contributory
8	72	no	Non-contributory
9	57	no	Non-contributory
10	54	no	Non-contributory
11	51	no	Non-contributory
12	86	no	Diabetes
13	55	yes-10/d	Non-contributory
14	63	no	Non-contributory
15	63	yes-20/d	Dafiro HCT, Atorvastin
16	70	no	Non-contributory
17	31	no	Non-contributory
18	46	yes-5/d	Nickel intolerance
19	75	no	Penicillin intolerance
20	66	no	Non-contributory
21	32	no	Non-contributory
22	72	no	Osteoporosis, hypertension
23	66	no	Hypertension
24	57	no	Non-contributory
25	55	no	Penicillin intolerance
26	69	no	Penicillin intolerance
27	78	no	Olmetec plus, Metohexal, Carmen, Adenuric
28	40	no	Penicillin intolerance
29	77	no	Tyronajod 75
30	52	no	Non-contributory
31	48	no	Nickel intolerance
32	70	no	Marcumar
33	55	no	Hypertension
34	45	no	Non-contributory
35	61	1 cigar/d	Non-contributory
36	47	no	Diabetes
37	74	no	Non-contributory
38	60	no	Non-contributory
39	54	no	Aspirin
40	57	no	Tyrox
41	93	no	Heart valve replacement
42	68	yes-20/d	Penicillin intolerance
43	38	no	Non-contributory
44	48	yes-30/d	Non-contributory
45	72	no	Cancer (Avastin), Oteoporoses, Hypertension (Bisopolol)
46	72	no	Hypertension (Losartan)
47	53	yes-10/d	Non-contributory
48	74	no	Non-contributory
49	47	yes-20/d	Non-contributory

**Table 2:** Characteristics of extracted teeth, reconstruction procedures and implants placed.

Patient Number	No. of Surgeries	No. of Implants	Implant Site	Periapical Infection	Previous RCT	Implant Type, Brand, Model	Implant Length, mm	Implant Diameter, mm	Immediate Loading	CTG	Cemented or Screwed Retention
1	2	1	21	yes	no	NobelReplace CC	13	3.5	no	yes	screwed
2	1	1	11	yes	yes	Straumann GM Helix	13	3.75	yes	yes	screwed
3	1	2	32	yes	no	NobelReplace CC	13	3.5	yes	yes	screwed
			42	no	no	NobelReplace CC	13	3.5	yes	yes	screwed
4	1	2	12	yes	yes	NobelReplace CC	16	3.5	yes	yes	screwed
			22	no	yes	NobelReplace CC	16	3.5	yes	yes	screwed
5	2	2	11	no	yes	NobelActive	15	4.3	yes	no	screwed
			22	no	yes	NobelActive	15	4.3	yes	no	screwed
6	1	1	12	no	yes	Nobel Speedy Groovy	13	3.3	no	yes	screwed
7	3	1	21	yes	no	Straumann GM Helix	13	4	yes	yes	cemented
8	1	1	12	no	yes	NobelReplace CC	13	3.5	yes	yes	screwed
9	1	2	21	yes	yes	Straumann GM Helix	13	4	yes	yes	screwed
			12	no	no	Straumann GM Helix	13	4	yes	yes	screwed
10	1	3	23	yes	no	NobelReplace CC	16	3.5	no	yes	screwed
			42	yes	no	NobelReplace CC	13	3.5	yes	no	screwed
			32	no	no	NobelReplace CC	13	4.3	yes	no	screwed
11	1	4	13	yes	yes	Straumann GM Helix	18	3.5	yes	yes	screwed
			12	yes	no	Straumann GM Helix	16	3.5	yes	yes	screwed
			22	yes	no	Straumann GM Helix	16	3.5	yes	yes	screwed
			23	yes	no	Straumann GM Helix	16	3.5	yes	yes	screwed
12	1	2	11	yes	no	Straumann GM Helix	16	3.5	yes	yes	screwed
			13	yes	no	Straumann GM Helix	11.5	4.3	yes	yes	screwed
13	1	1	21	yes	no	Straumann GM Helix	13	3.75	yes	yes	screwed
14	1	1	21	yes	yes	Straumann GM Helix	13	3.75	yes	yes	screwed
15	1	1	21	yes	no	NobelReplace CC	13	4.3	yes	yes	screwed
16	1	1	21	yes	yes	NobelReplace CC	13	4.3	no	yes	screwed
17	1	2	11	yes	yes	NobelReplace CC	16	3.5	yes	yes	screwed
			21	no	no	NobelReplace CC	16	3.5	yes	yes	screwed
18	2	2	11	yes	yes	NobelReplace CC	13	3.5	no	no	screwed
			22	yes	no	NobelReplace CC	13	3.5	no	no	screwed
19	2	1	22	yes	yes	NobelActive	15	3.5	no	yes	screwed
20	1	1	13	yes	yes	NobelReplace CC	16	3.5	no	yes	screwed
21	2	1	12	yes	yes	NobelReplace CC	16	3.5	no	yes	screwed
22	1	2	43	no	yes	NobelReplace CC-PMC	13	3.5	no	yes	screwed
			41	no	no	NobelReplace CC-PMC	13	3.5	no	yes	screwed
23	2	1	11	yes	yes	NobelReplace CC	16	3.5	yes	yes	cemented
24	2	1	11	yes	no	NobelActive	13	3.5	no	yes	cemented
25	1	1	43	yes	no	NobelReplace CC	13	4.3	yes	no	screwed
26	2	2	13	yes	yes	NobelReplace CC	13	4.3	no	no	cemented
			23	yes	yes	NobelReplace CC	16	4.3	no	no	screwed
27	1	1	11	yes	yes	NobleParallel CC	15	3.75	yes	yes	cemented
28	2	1	21	yes	yes	NobelReplace CC	11.5	3.5	no	yes	screwed
29		1	22	yes	no	GM Helix	18	4.3	no	yes	screwed
30	2	1	21	yes	yes	NobleParallel CC	16	3.5	no	yes	screwed
31	1	1	21	yes	yes	NobelReplace CC	13	3.5	yes	yes	cemented
32	1	1	21	yes	yes	Straumann GM Helix	16	3.75	yes	yes	screwed
33	2	1	21	yes	yes	NobelReplace CC	11.5	3.5	no	yes	cemented
34	1	1	23	yes	no	NobleParallel CC	13	3.75	no	yes	screwed
35	1	1	21	yes	no	NobelReplace CC	13	3.5	no	yes	screwed
36	1	1	12	yes	no	Straumann GM Helix	13	3.5	yes	yes	screwed
37	1	1	22	yes	yes	Straumann GM Helix	13	3.5	yes	yes	screwed
38	1	1	11	yes	yes	NobelActive	15	3.5	yes	yes	cemented
39	1	1	41	yes	yes	NobelReplace CC	16	3.5	yes	yes	screwed
40	1	1	21	yes	yes	Straumann GM Helix	13	3.75	no	yes	screwed
41	1	1	22	yes	yes	NobelReplace CC	13	4.3	yes	no	screwed
42	2	2	21	yes	yes	Straumann GM Helix	13	3.75	yes	yes	screwed
			13	yes	yes	Straumann GM Helix	16	4	no	no	screwed
43	2	1	11	yes	yes	NobelReplace CC	13	3.5	no	yes	screwed
44	1	1	11	yes	yes	NobleParallel CC	13	3.75	yes	yes	screwed
45	1	1	21	yes	no	GM Helix	16	3.75	yes	yes	screwed
46	1	1	21	yes	yes	NobleParallel CC	13	3.75	yes	yes	screwed
47	2	1	21	yes	no	NobleParallel CC	13	3.75	no	yes	screwed
48	2	1	11	yes	no	Neodent-Straumann CM Alvim	16	3.5	no	yes	screwed
49	1	2	12	no	no	Straumann BLX	14	3.75	yes	no	screwed
			22	yes	no	Straumann BLX	14	3.75	yes	no	screwed

RCT = root canal treatment; CTG = connective tissue graft.

**Table 3:** List of postoperative complications occurred.

Patient No.	Site No.	Complication Type
5	11	Peri-implantitis
17	11	A little piece of the lamina removed 18 months after surgery
33	21	Palatal cyst developed four years after surgery
48	11	Fracture of the provisional crown

**Table 4:** Synthetic table of potentially influencing factors and incidence of complications.

Potentially Influencing Factor		N. of Implants	N. of Complications	Significance (*p*-Value)
Smoking status	Smoker	18	1	0.43
	Non-smoker	47	3	
Periapical infection	Yes	53	3	0.42
	No	12	1	
Previous root canal treatment	Yes	36	3	0.31
	No	29	1	
Prosthetic connection	Screwed	57	3	0.35
	Cemented	8	1	
Location	Maxilla	57	4	0.58
	Mandible	8	0	
Loading mode	Immediate	40	2	0.35
	Delayed	25	2	

## Data Availability

Data can be made available if requested.
